# Caspase activity and apoptotic signaling in proliferating C2C12 cells following cisplatin or A23187 exposure

**DOI:** 10.1016/j.dib.2016.03.032

**Published:** 2016-03-12

**Authors:** Darin Bloemberg, Joe Quadrilatero

**Affiliations:** Department of Kinesiology, University of Waterloo, Waterloo, Ontario, Canada

## Abstract

Investigating cell death signaling using cell culture is commonly performed to examine the effects of novel pharmaceuticals or to further characterize discrete cellular signaling pathways. Here, we provide data regarding the cell death response to either cisplatin or A23187 in sub-confluent C2C12 cells, by utilizing several concentrations and incubation times for each chemical. These data include an assessment of the activation of the proteolytic enzymes caspase-3, caspase-8, caspase-9, calpain, and cathepsin B/L. Additionally, the expression of the apoptosis-regulating proteins Bax, Bcl2, and p53 are presented.

**Specifications table**

**Table:** 

Subject area	*Biology*
More specific subject area	*Apoptosis*, *caspases*
Type of data	*Graphs and Figures*
How data was acquired	*Spectrofluorometry*, *immunoblotting*, *microscopy*
Data format	*Analyzed*
Experimental factors	*C2C12 cells were treated with cisplatin or A23187*
Experimental features	*Cells were administered cisplatin or A23187 and collected at several time points. They were then prepared for spectrofluorometric assessment of proteolytic enzyme activity or were analyzed for apoptosis-related protein expression using immunoblotting*. *Brightfield microscope images were also acquired for qualitative assessment of cell morphology*.
Data source location	*University of Waterloo*, *Waterloo*, *Ontario*, *Canada*
Data accessibility	*All data are provided with this article*

**Value of the data**

• The data describe the cell death response in proliferating C2C12 cells following exposure to several concentrations and incubation periods with either cisplatin or A23187.
• Provides data regarding the specific pathways of cell death activation in C2C12 cells to either cisplatin or A23187.
• The data demonstrate that cell death in C2C12 cells by cisplatin involves significant activation of p53 and caspases, while A23187 involves caspase-independent mechanisms.

## 1 Data

**Fig. 1:**
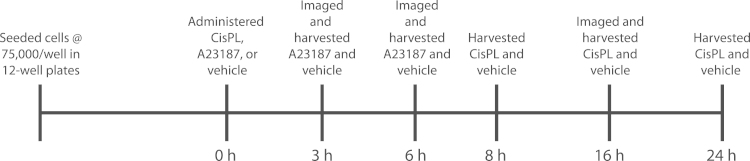
Overview of experimental treatment protocol.

Two key signals which regulate the induction of apoptosis are DNA damage and calcium (Ca^2+^) [1], [2]. Despite the common use of cisplatin (CisPL) and Ca^2+^ ionophores such as A23187 to induce apoptosis in cell culture experiments, limited evidence exists in C2C12 cells. Here, we present data describing the cell death response in sub-confluent C2C12 cells exposed to CisPL or A23187 (Fig. 1).

### 1.1 CisPL-induced apoptotic signaling in C2C12 cells

**Fig. 2:**
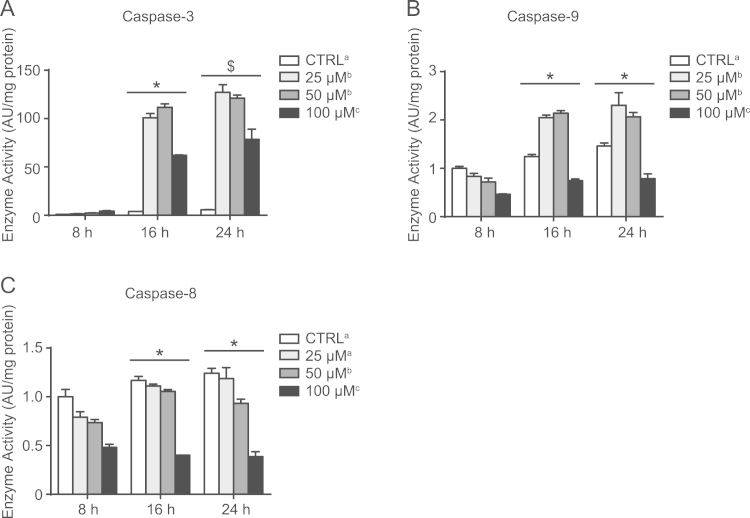
Caspase activity in response to CisPL treatment. (A) CisPL induced concentration- and time-dependent changes in caspase-3 activity. (B) Similar effects were observed for caspase-9. (C) CisPL administration did not elevate the activity of caspase-8. Values are expressed relative to CTRL (vehicle-treated) 8 h, which has been arbitrarily given a value of 1.0. *p<0.05, significantly different than 8 h (main effect of time); $*p*<0.05, significantly different than 8 h and 16 h (main effect of time). Main effects (*p*<0.05) for CisPL dose are indicated with superscript letters, where groups with different letters are significantly different from each other.

**Fig. 3:**
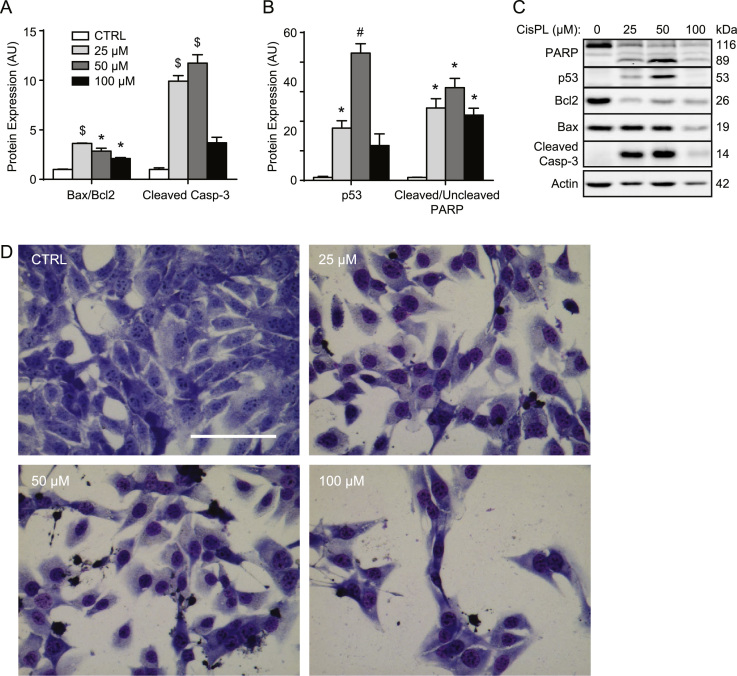
Changes to expression of apoptotic signaling proteins in response to CisPL at the 16 h time point. (A) All CisPL treatments elevated the Bax/Bcl2 ratio, while 25 µM and 50 µM doses significantly increased cleaved caspase-3 levels. (B) CisPL elevated p53 protein content and the relative amount of cleaved PARP. (C) Representative immunoblots. (D) Brightfield microscope images of Giemsa stained CTRL (vehicle-treated) and CisPL treated cells. Values for CisPL-treated groups are expressed relative to CTRL groups, which have been arbitrarily given a value of 1.0. *p<0.05 compared to CTRL, $*p*<0.05 compared to CTRL and 100 µM, #*p*<0.05 compared to all other groups. Scale bar represents 100 µm.

Beginning with the previously used concentrations [3], [4], C2C12 cells were administered CisPL in increasing doses and intermittently collected over a period of 24 h (Fig. 2, Fig. 3). Caspase activity was spectrofluorometrically measured using fluorogenic substrates specific for each enzyme [5], [6]. CisPL treatment caused time-dependent increases (*p*<0.05) in the activity of caspase-3 and caspase-9 (Fig. 2A and B). For caspase-3 and caspase-9, 25 µM and 50 µM CisPL induced larger (*p*<0.05) elevations in enzyme activity than 100 µM (Fig. 2A and B). However, despite increased (*p*<0.05) caspase-8 activity at 16 h and 24 h compared to 8 h, 50 µM and 100 µM CisPL doses reduced (*p*<0.05) caspase-8 enzyme activity (Fig. 2C). Data regarding the levels of apoptosis-regulating proteins at the 16 h time point also indicated concentration-dependent changes (Fig. 3). Here, CisPL elevated (*p*<0.05) the Bax/Bcl2 ratio, the amount of cleaved caspase-3, p53 protein levels, and the ratio of cleaved/uncleaved PARP protein (Fig. 3A–C). Of note, 50 µM CisPL dramatically increased (*p*<0.05) p53 protein content above that caused by other concentrations. Despite observing the most significant changes to apoptotic markers with 25 µM and 50 µM CisPL, qualitative assessment of brightfield microscope images of Giemsa stained cells indicated that 100 µM had the greatest negative impact on cell confluence and morphology (Fig. 3D), perhaps suggesting non-apoptotic mechanisms of cell death at this dose.

### 1.2 A23187-induced cell death signaling in C2C12 cells

**Fig. 4:**
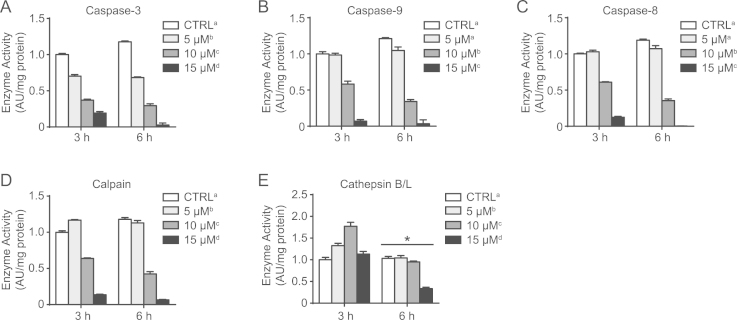
Proteolytic enzyme activity induced by A23187. A23187 had concentration-dependent but time-independent effects on the activity of caspase-3 (A), caspase-9 (B), caspase-8 (C), and calpains (D), where 10 µM and 15 µM A23187 generally reduced enzyme activities. (E) Cathepsin B/L activity was higher at the 3 h time point. 5 µM and 10 µM induced cathepsin B/L activity and 15 µM reduced cathepsin B/L activity. Values are expressed relative to CTRL (vehicle-treated) 3 h, which has been arbitrarily given a value of 1.0. *p<0.05, significantly different than 3 h (main effect of time). Main effects (*p*<0.05) for A23187 dose are indicated with superscript letters, where groups with different letters are significantly different from each other.

**Fig. 5:**
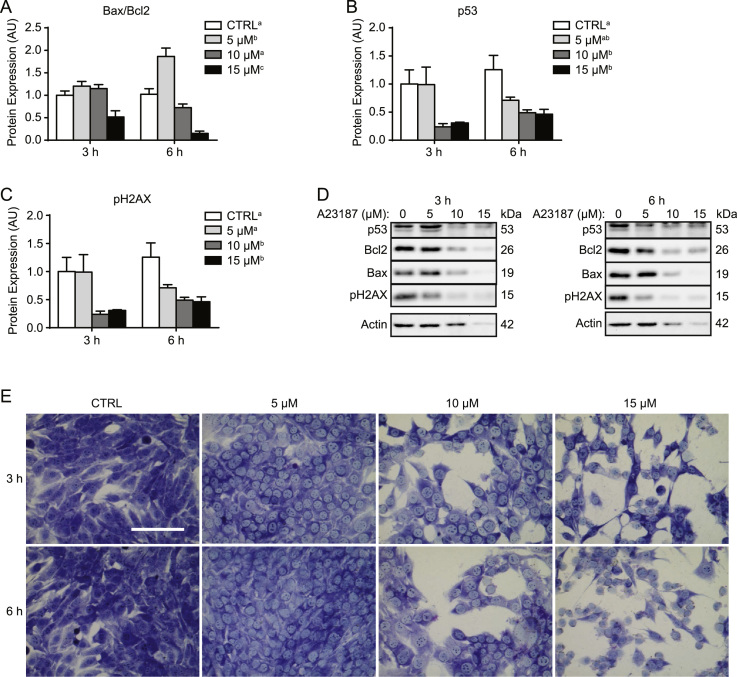
Changes to expression of apoptotic signaling proteins in response to A23187. A23187 treatments had concentration-dependent effects on the Bax/Bcl2 ratio (A), p53 (B), and pH2AX (C). (D) Representative immunoblots. (E) Brightfield microscope images of Giemsa stained CTRL (vehicle-treated) and A23187 treated cells. Values are expressed relative to CTRL 3 h, which has been arbitrarily given a value of 1.0. Main effects (*p*<0.05) for A23187 dose are indicated with superscript letters, where groups with different letters are significantly different from each other. Scale bar represents 100 µm.

Sustained high levels of cytosolic Ca^2+^ can activate apoptotic signaling mechanisms [7]. While several ways of mimicking ER/Ca^2+^-stress exist, ionophores allow specific alterations to ion levels without affecting accessory cellular protein functions. A23187 is a partially-selective Ca^2+^ ionophore widely used to increase cytosolic Ca^2+^ levels in cell culture. Previously, 1 µM A23187 treatment for 2 h was shown to elevate calpain activity 3-fold in proliferative C2C12 cells, while increasing concentrations caused progressive drops in cell viability over 6 h [8]. Here, varying concentrations of A23187 were administered to cells over 6 h in order to assess the appropriate conditions for causing Ca^2+^-induced apoptotic signaling in sub-confluent C2C12 cells. These data demonstrate that A23187 treatment did not cause caspase-3, −8, or −9 activation at either time point (Fig. 4A–C). In fact, 10 µM and 15 µM doses generally reduced (*p*<0.05) the activity of these three proteolytic enzymes (Fig. 4A–C). While 5 µM A23187 slightly elevated (*p*<0.05) calpain activation (Fig. 4D), two higher concentrations reduced (*p*<0.05) calpain enzyme activity (Fig. 4D). Assessing the lysosomal hydrolase cathepsin B/L indicated that activity was generally higher (*p*<0.05) at 3 h compared to 6 h, where 5 µM and 10 µM doses increased (*p*<0.05) activity, while 15 µM reduced (*p*<0.05) activity, particularly at the 6 h time point (Fig. 4E). Finally, 5 µM A23187 appeared to moderately activate upstream apoptotic signaling as indicated by an elevated (*p*<0.05) Bax/Bcl2 ratio (Fig. 5A and D). However, higher concentrations reduced (*p*<0.05) the Bax/Bcl2 ratio, p53 protein (Fig. 5B and D), and levels of pH2AX (Fig. 5C and D), a marker of DNA damage. Despite this relative lack of apoptotic signaling activation, brightfield microscope images of Giemsa stained cells demonstrated dramatic impacts on cell morphology caused by 10 µM and 15 µM A23187 compared to vehicle-treated CTRL cells (Fig. 5E).

## 2 Experimental design, materials and methods

### 2.1 Cell culture and experiment

C2C12 mouse skeletal muscle myoblasts (ATCC) were cultured as previously performed [6], [9] on polystyrene cell culture plates (BD Biosciences) with media consisting of low-glucose DMEM (ThermoFisher) with 10% fetal bovine serum (FBS; ThermoFisher) and 1% penicillin/streptomycin (ThermoFisher). Upon reaching 60–70% confluence, cells were administered either cisplatin (CisPL; Enzo Life Sciences) or the calcium ionophore A23187 (Sigma Aldrich), or maintained in a control (CTRL) condition which was given an equal volume of vehicle (saline for CisPL experiments; DMSO for A23187 experiments). For CisPL experiments, cisplatin was dissolved fresh in sterile saline (0.9% NaCl) at 3 mM, diluted in warmed culture media to working concentrations of 25 µM, 50 µM, or 100 µM, and administered to cells for 8 h, 16 h, or 24 h (Fig. 1). A23187 was dissolved in DMSO at a concentration of 1 mM, stored at −20 °C, and diluted in warmed culture media to working concentrations of 5 µM, 10 µM, or 15 µM before being administered to cells for 3 h or 6 h (Fig. 1).

### 2.2 Preparation of cell lysates

At the indicated time points, cells were collected via trypsinization, centrifuged at 1000*g*, and stored at −80 °C. For enzyme activity assays, cell lysates were generated by sonicating cells in lysis buffer containing 20 mM HEPES, 10 mM NaCl, 1.5 mM MgCl, 1 mM DTT, 20% glycerol, and 0.1% Triton-X100 at a pH of 7.4. Separate cells were prepared for immunoblotting using lysis buffer containing protease inhibitors (Complete Cocktail, Roche). Protein content of cell lysates was determined using the BCA protein assay method.

### 2.3 Proteolytic enzyme activity

Enzymatic activity of caspase-3, −8, −9, cathepsin B/L, and calpain was measured with the fluorogenic substrates Ac-DEVD-AMC, Ac-IETD-AMC, Ac-LEHD-AMC, Z-FR-AFC, and Suc-LLVY-AMC (Enzo Life Sciences), respectively [5], [6]. Calpain activity was determined by accounting for the reduction in fluorescence caused by the calpain inhibitor Z-LL-CHO (Enzo Life Sciences). Cell lysates were incubated in duplicate in black 96-well plates with the appropriate substrate at room temperature, 30 °C, or 37 °C for caspase, cathepsin, and calpain analyses, respectively. Fluorescence was measured using a SPECTRAmax Gemini XS microplate spectrofluorometer. Enzyme activity was normalized to total protein content and expressed as fluorescence intensity in arbitrary units (AU) per milligram protein.

### 2.4 Immunoblotting

Immunoblotting was performed as previously described [6], [9], [10]. Samples were loaded with equal amounts of protein (30 µg) and separated by SDS/PAGE before being transferred onto PVDF membranes. Membranes were probed with primary antibodies against Bcl2 (1:200), Bax (1:1000), PARP (1:500), pH2AX (1:500), p53 (1:500) (Santa Cruz Biotechnology), cleaved caspase-3 (1:1000), or actin (1:2000) (Sigma Aldrich) overnight at 4 °C and then incubated with the appropriate horseradish peroxidase (HRP)-conjugated secondary antibody (Santa Cruz Biotechnology) for 1 h at room temperature. Bands were visualized using the Clarity ECL blotting substrate (Bio-Rad) and the ChemiGenius 2 Bio-Imaging System (Syngene). The approximate molecular weight for each protein was estimated using Precision Plus Protein WesternC Standards and Precision Protein Strep-Tactin HRP Conjugate (Bio-Rad).

### 2.5 Microscopy

Giemsa staining was performed to assess changes to cell morphology [11]. Briefly, cells were fixed in ice-cold methanol for 10 min, allowed to air-dry, and incubated with 1:20 Giemsa solution (Sigma Aldrich) for 45 min at room temperature. Images were acquired using a Nikon microscope equipped with a PixeLink digital camera.

### 2.6 Statistics

Results are presented as means±SEM, where *n*=3. For comparisons across time points, 2-way ANOVAs were performed with Tukey post-hoc analyses to detect main effect differences. For comparisons at individual time points in Figs 3, 1-way ANOVAs were performed with Tukey post-hoc analyses to detect differences between treatment groups. In all cases, *p*<0.05 was considered statistically significant.
